# Promoting Behavioral Change to Improve Health Outcomes

**DOI:** 10.3390/bs15040417

**Published:** 2025-03-25

**Authors:** John A. Parkinson

**Affiliations:** Wales Centre for Behaviour Change, Department of Psychology, Bangor University, Gwynedd LL57 2AS, UK; j.parkinson@bangor.ac.uk

## 1. Introduction

The key to long-term mental and physical health, including a high quality of life without the burden of disease, is adopting and sustaining behaviors that prevent illness and promote resilient health. However, the prevalence of non-communicable diseases is increasing across the world, including obesity, type 2 diabetes, cardiovascular disease, mental health disorders, etc. Such a burden is unsustainable and reduces quality of life, productivity, and wellbeing across the board (Edwards, 2024; Olshansky, 2018). In the UK, where healthcare is free at the point of delivery, the National Health Service is struggling to manage this increased burden of disease across citizens (DHSC, 2018). Increasingly, arguments are being made for an emphasis on prevention and an understanding of the potential return on investment of acting early to prevent or reduce illness and promote wellbeing (Edwards, 2024; Masters et al., 2017; Musich et al., 2016).

By zooming out and viewing this health crisis from a distance, it seems clear that individuals, groups, and even entire populations need to change their behavior in order to promote better health outcomes. Adopting preventative habits, such as eating a healthy diet, exercising regularly, developing a healthy sleeping habit, and reducing or cutting out drug use (tobacco, alcohol, etc.), the burden of disease would be diminished, and the number of years a person lives disease-free (healthspan) would increase significantly. Currently, the opposite trend is true for many societies (Davoud¡-Monfared et al., 2024; DHSC, 2018; Wagner & Brath, 2012).

Sustained behavior change is challenging, not only because our good intentions are not always followed by good actions (Allan et al., 2008; Gould et al., 2024; Verplanken & Orbell, 2022). We have seemingly evolved with multiple brain mechanisms that make decisions and control our behavior in ways that are not always consistent or outwardly rational (Khalil & Amin, 2023; Lalani et al., 2023). As such, the particular value of behavioral science is in recognizing and understanding that non-conscious behavioral drivers can sometimes override deliberate intentions. Factors such as social norms, habits, and contextual elements—including physical architecture—play a crucial role in shaping behavior, often leading individuals to act contrary to their explicit intentions (Kahneman, 2013; Thaler & Sunstein, 2009).

This discrepancy between intention and action, commonly referred to as the “intention–action gap”, is well-documented in the literature and can be traced back at least as far as Plato’s *Republic*. A dual-process framework provides a theoretical lens through which this gap can be understood, suggesting that behavior is governed by two interacting pathways: one intuitive and automatic, and the other deliberative and controlled (Evans & Stanovich, 2013). Typically, these systems operate in concert to guide behavior, balancing automatic responses with intentional regulation. However, in certain contexts, these systems may come into conflict, and in such cases, Type 1 processes—characterized as evolutionarily older and more directly linked to behavioral execution at a neural level—are likely to dominate decision-making and action (Levant & Parkinson, 2014). Furthermore, with this insight, contemporary approaches to behavior change are more nuanced and cleverly designed to target the right systems for the right type of behavior. For example, the COM-B model, developed from Michie’s Behavior Change Wheel (Cane et al., 2012; Michie et al., 2011) considers behavior through the lenses of capability, opportunity and motivation, acknowledging the myriad influences across contexts. Building upon this complexity, there is a growing recognition that system approaches are required to give a holistic view of how behavior is determined by the larger system within which it sits. This recognizes the importance of not only intervention at different levels (Bioethics, 2007; Chater & Loewenstein, 2023) but also understanding unintended consequences of interventions, as well as the need to map actors and behaviors within larger systems to fully understand the drivers and influencing factors (Gould et al., 2024; Parkinson et al., 2025; Reynolds, 2024).

Within this context, this Special Issue sought to bring together research that explored health-related behavior change, underpinned by contemporary theory, with the aim of promoting longer-term wellbeing and health across a number of domains and with a diversity of methodologies.

## 2. Research by Health Domain

The papers in this Special Issue span a diversity of domains and behaviors, demonstrating the far-reaching value of a behavioral science approach. For example, three studies focused on substance use. One used a brief motivational intervention to reduce excessive drinking in students (contribution 1) incorporating motivational interviewing and systematic motivational counseling. The intervention was successful, though there were gender differences around the specificity of efficacy for the interventions. The second study explored users’ perceptions of therapeutic services for substance abuse to inform better experiences and outcomes for those enrolled (contribution 2). Users perceived the therapeutic context as distinct from the ‘real world’, and the authors emphasized the need for agency and empowerment to support the recovery process. The third study evaluated a novel acceptance and commitment therapy (ACT) intervention for substance users to better understand the process of change and recovery (contribution 3). The intervention was co-designed by people in recovery, and success was related to core ACT mechanisms at play.

In the context of physical exercise, one study focused on exercise intentions from the perspective of job-related factors (contribution 4), and identified certain high-risk groups. Recommendations for comprehensive work-based support were made. A second study considered exercise and its relationship to sleep quality with the added perspectives of self-control and mindfulness (contribution 5). As expected, exercise predicted sleep quality in college students, and the authors went on to explore the mediation and moderation of this relationship with self-control and mindfulness. Two studies had diet and weight management as a health focus. Shourche et al. (contribution 6) tested the Life Advancement and Enhancement Program (LEAP) in a group of overweight females. The results showed that adding LEAP to a weight management practice had a significant effect on improving outcomes. Exploring a different avenue, Halabi et al. (contribution 7) considered non-dairy alternatives in coffee drinkers from the perspective of health awareness and consumer’s beliefs about the consequences of their actions. Based on the findings, the researchers made recommendations within the context of the Behavior Change Wheel framework, relating to fostering inclusivity, health awareness, and supporting environmental sustainability efforts.

Several studies considered medical adherence and/or the use of augmented medical devices. For example, one study sought to better understand home medication management practices in order to improve medical adherence (contribution 8) and proposed personalized guidance to patients from their healthcare providers. A second study explored attitudes and behaviors, including negative emotions such as fear and anxiety, towards vaccinations using a multicultural network analysis (contribution 9). A clear relationship was found between vaccination status and cognitive symptoms of fear and anxiety, suggesting novel routes for intervention to promote vaccine acceptance. Another study considered user intentions related to wearing augmented hearing aid devices (contribution 10) and found that functionality quality, perceived interaction speed, and perceived usability related to confidence in use of the novel devices.

Three studies focused on mental health. One looked at the role of guilt in mental health ‘burnout’ (contribution 11) and found that feelings of guilt appeared to drive psychosomatic symptoms during the burnout process. They recommend that future studies of burnout should encompass emotions including guilt as potentially important elements of the process. Two other studies focused on COVID-19. The first used a longitudinal design to track the mental health of university students over a two-year period during and after the initial COVID-19 epidemic (contribution 12). They found that distress and generalized anxiety remained high following the pandemic and argue that student mental health ‘is still in crisis’ with a need for further support for this cohort who are transitioning into the world of work. Another study looked at the potential role of ‘behavioral fatigue’ during the acute phase of COVID-19 (contribution 13) using a COM-B analysis and concluded that in order to maintain prolonged behavior change, capabilities, opportunities and motivation are all important for target behaviors. Continuing in the context of COVID-19, one experimental study explored risk behaviors from the perspective of psychological distance, testing an intervention designed to make COVID-19 feel more concrete and real (contribution 14). The authors demonstrated that participant risk perceptions were influenced by psychological distance (whether something is close/concrete or far/abstract) and were able to reduce the willingness of participants to engage in risky COVID-19 behaviors by making the concept of the COVID-19 virus more concrete.

To round off the diversity of behavioral domains, Hutchings et al. (contribution 15) evaluated data from an RCT trial of the KiVa anti-bullying school-based program in the UK. The results supported a social architecture model of bullying behavior and suggested further work to refine the model in addressing bullying as a public health challenge. Finally, Wetton et al. (contribution 16) used behavioral systems mapping to understand sustainability and waste management behaviors in Kenya. They developed a map which demonstrated key actors, sub-systems, and feedback loops that helped explain poor solid waste management practices and could be used to create systemic behavior change interventions in the future.

## 3. Research by Methodology and Approach

Across this Special Issue, two papers focused explicitly on prevention: one testing an intervention to reduce bullying, and the other exploring ways to promote COVID-19-safe behaviors (contribution 14; contribution 15). Additionally, eleven papers sought to use contemporary analyses in order to better understand a problem situation or behavior (contribution 2; contribution 4; contribution 5; contribution 7; contribution 8; contribution 9; contribution 10; contribution 11; contribution 12; contribution 13; contribution 16). Finally, three focused specifically on secondary prevention (Simeonsson, 1991) for either substance use or weight management (contribution 1; contribution 3; contribution 6).

Furthermore, of the approaches above, three papers based their study on the COM-B framework (contribution 7; contribution 13; contribution 16), whilst seven used questionnaires followed by a variety of modeling techniques, such as structural equation modeling (contribution 4; contribution 5; contribution 8; contribution 9; contribution 10; contribution 11; contribution 12). Finally, four employed intervention designs that were based on specific psychological (contribution 6; contribution 14; contribution 15) or therapeutic theories (contribution 1). Of these, only one paper explicitly incorporated dual-process theory (contribution 14).

## 4. Conclusions

This Special Issue showcases novel and impactful original research within the domain of promoting healthy behavior change. First and foremost, the research demonstrates the value of an approach based on behavioral science, particularly in demonstrating the value of integrating psychological theory, such as the dual-process approach (Evans & Stanovich, 2013), in developing interventions. The work herein also demonstrates the value of employing contemporary frameworks, such as COM-B (Michie et al., 2011), and testing them in the real world (Atkins et al., 2017).

**Figure 1:**
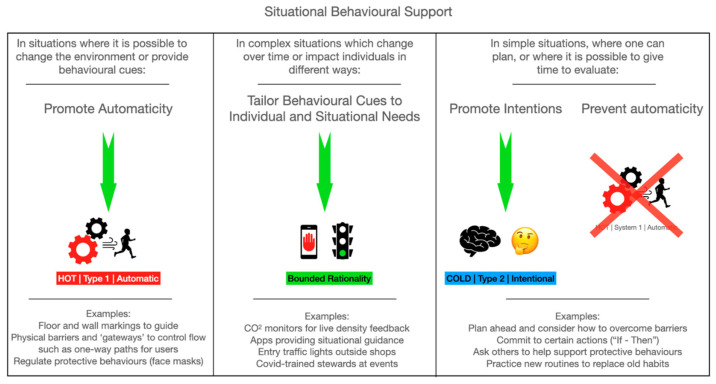
Taken from (Gould et al., 2024).

Recently, we developed a high-level tool based on an integration of COM-B and dual-process theory ((Evans & Stanovich, 2013; Michie et al., 2011) to support health and wellbeing during the COVID-19 pandemic. The aim was to help those designing interventions to consider whether to target automatic (Type 1) or cognitive (Type 2) processes, and whether to focus on capability, opportunity, or motivation (see Figure 1, taken from (Gould et al., 2024). It also emphasizes that the moment of decision-making is often when behavior is most susceptible to influence. We hope that such an approach can improve the efficacy of behavioral interventions to promote positive and long-lasting health outcomes.

Ultimately, the combination of knowledge dissemination, targeted behavior change interventions, supportive environments, and strategic policies can lead to sustainable behavior change. The benefits of these interventions are twofold: individuals lead healthier, longer lives; and the societal costs associated with treating preventable diseases are considerably reduced. The field of health economics is becoming increasingly important in this regard, identifying the financial (and other) return on investment of implementing preventative and health-promoting interventions to build more human and social resilience to health challenges (Edwards, 2024; Whiteley et al., 2024). There is currently a paucity of research focused on identifying and utilizing dual-process theory in order to leverage the most effective behavior change techniques (Gould et al., 2024; Parkinson et al., 2014), particularly with regard to actually measuring behavioral outcomes rather than proxies such as attitudes or intentions (Murphy et al., 2023). Finally, given the complex nature of health challenges, future research will need to consider whole-system approaches, including system thinking, as well as considering at what level within a system an intervention is likely to have the largest benefit (Chater & Loewenstein, 2023; Parkinson et al., 2025; Reynolds, 2024).
